# Hypomagnesemia and Acute Cognitive Decline in Older Adults: An Evaluation of Clinical Practice and Cognitive Outcomes at a National Health Service (NHS) Trust in England

**DOI:** 10.7759/cureus.88920

**Published:** 2025-07-28

**Authors:** Xuan Ning Lai, Vasvi Sadhwani, Wen Min Ng, Akif Gani

**Affiliations:** 1 General Medicine, Newcastle upon Tyne Hospitals, Newcastle upon Tyne, GBR; 2 Acute Internal Medicine, Wexham Park Hospital, Slough, GBR

**Keywords:** acute, cognitive decline, delirium, elderly, electrolyte, electrolyte disturbance, geriatric, hypomagnesemia, magnesium, older adults

## Abstract

Introduction

Magnesium is essential for regulating cardiovascular, neuromuscular, and respiratory functions. Hypomagnesemia in older adults is often overlooked and insufficiently managed. Inadequate monitoring and correction of hypomagnesemia may leave old and frail patients more vulnerable to acute cognitive decline, which in some cases can be preventable.

This study assessed the current management of hypomagnesemia in older adults admitted to the geriatric wards of an NHS Trust and its association with acute cognitive decline, which is defined in this study as any sudden deterioration from baseline cognition, including delirium or new-onset confusion, identified using the 4-AT tool (score ≥4) and collateral history. The 4AT Rapid Clinical Test for Delirium Detection encompasses four components, namely Alertness, Abbreviated Mental Test-4, Attention and Acute change or fluctuating course.

Methods

A retrospective review of old and frail patients admitted to geriatric wards across two hospital sites over a month was conducted. Patients aged 65 years or above and those aged between 55 to 64 with clinical frailty were included. Electronic records were used to compare acute cognitive outcomes in patients with hypomagnesemia and those with normal magnesium levels. Multivariate analysis was performed to assess the predictors of acute cognitive impairment.

Results

Of the 667 hospitalized older adult patients included, 149 (22.3%) had hypomagnesemia, while 518 (77.7%) had normal levels. Among the 149 patients, 18 (12.2%) had moderate-to-severe deficiency (≤0.5 mmol/L); of these, 27.8% received intravenous supplementation, 38.9% received oral supplementation, and 33.3% received no treatment. Among the remaining 131 patients with mild hypomagnesemia, 34.4% received some form of supplementation, while 65.5% had none. After the check-up at admission, only 40.3% of all hypomagnesemic patients had their serum magnesium levels checked at intervals recommended by the trust guideline.

In the multivariable logistic regression model, after adjusting for age, sex and potential clinical confounders, i.e. infection, electrolyte disturbances including hypocalcemia, hypercalcemia, hyponatremia and hypernatremia, acute kidney injury, pain, acute stroke, and constipation, patients with hypomagnesemia had 2.35 times greater odds of developing acute cognitive deterioration (OR (Odds ratio) =2.354; 95% CI (Confidence interval): 1.543-3.604; p<0.001).

These findings suggest the independent association between hypomagnesemia and cognitive decline, underscoring the need for its improved recognition and management in clinical practice.

Conclusion

Hypomagnesemia may be a significant contributor to acute cognitive impairment in old and frail patients.

## Introduction

Magnesium plays a vital role in human physiology, which ranges from regulating cardiovascular, neuromuscular, and respiratory function to supporting bone integrity, mental health, and cognitive function [1]. Despite its importance, hypomagnesemia remains a frequently underdiagnosed electrolyte disturbance in patients, especially the geriatric population, with implications extending beyond the commonly recognized neuromuscular and cardiovascular effects [2,3]. It is likely often overlooked due to its typically subtle symptoms until levels become severely depleted, as well as a limited understanding of magnesium physiology among healthcare providers [4]. A study by Kim et al. in an intensive care setting suggests that magnesium deficiency may be a key yet often poorly recognized contributor to acute cognitive decline, defined in this study as any sudden deterioration from baseline cognition, including delirium or new-onset confusion, which has significant consequences on patient well-being and outcomes as well as healthcare costs [5,6]. However, adherence to established monitoring and treatment protocols is inconsistent, leaving older adults susceptible to potentially preventable acute cognitive decline.

This study evaluated the adherence of clinicians at an NHS Foundation Trust in England to the local treatment and monitoring guidelines of adult hypomagnesemia in old and frail patients, as well as explored the significance of hypomagnesemia as a contributor to acute cognitive decline. The trust defines normomagnesemia as serum magnesium levels of 0.7-1.0 mmol/L, and hypomagnesemia is classified as levels below 0.7 mmol/L [7]. While severity can be stratified (mild: 0.51-0.69 mmol/L; moderate: 0.31-0.5 mmol/L; severe: ≤0.3 mmol/L), this study does not focus on the severity, given the poor correlation between serum magnesium and the total body stores [7]. Old and frail adults face increased susceptibility due to age-related reductions in dietary intake, intestinal absorption, and renal conservation of magnesium [1]. Prior research also suggests that even mild magnesium deficits may contribute to cognitive dysfunction in the elderly, often being attributed as typical age-related decline [1].

The geriatric wards in this NHS Trust care for a large cohort of frail, multimorbid patients. However, compliance with institutional management guidelines and its potential relevance to cognition has not been systematically evaluated earlier. This study addresses two critical objectives: (1) to assess clinician adherence to local hypomagnesemia protocols, and (2) to establish the strength of association between magnesium deficiency and acute cognitive decline in this cohort. This study aims not only to assess gaps in current care but also identify the clinical relevance of magnesium in maintaining cognitive function.

## Materials and methods

Subjects and study design

This retrospective study included 667 old and frail patients who had been inpatients in the geriatric wards of Newcastle upon Tyne Hospitals NHS Foundation Trust in England across two hospital sites, namely the Freeman Hospital and the Royal Victoria Infirmary, between January 5 and February 5, 2025. Data were collected across a total of nine wards. Patients were eligible for inclusion if they were admitted under geriatric care within the trust during the study period and had at least one recorded serum magnesium level during their admission. While the majority were aged 65 years or older, patients aged 55 to 65 years were also included where clinical judgement had warranted geriatric admission based on frailty, multimorbidity, cognitive impairment, or functional decline, as allocation to geriatric care in this trust is based on clinical complexity rather than age alone. Frailty was evaluated using the Rockwood clinical frailty scale (CFS) [8] across all patients who were assessed for geriatric admission, including those aged 55-64, with frailty being defined as a CFS score of five or higher.

Patients were excluded if they lacked any documented serum magnesium measurement during admission, were admitted under non-geriatric specialties, were less than 55 years old, or had insufficient clinical records to allow retrospective assessment of their cognitive status. These exclusion criteria were applied to maintain data quality and ensure reliable outcome assessment.

Data on age, sex, initial and follow-up magnesium levels, treatment received, and presence of acute cognitive decline were gathered from the electronic hospital records.

Analysis of serum magnesium levels

All included patients underwent at least one serum magnesium measurement during their hospital admission, irrespective of subsequent monitoring. Consistent with the trust's established criteria, normomagnesemia was defined as serum levels between 0.7-1.0 mmol/L, while hypomagnesemia was classified as values ≤0.69 mmol/L [6]. Based on these parameters, the cohort was divided into two distinct groups: 149 patients demonstrated hypomagnesemia on one or more occasions during the observation period, compared to 518 patients who had normal magnesium levels throughout. Severity was noted as the trust's treatment guideline differs between mild and moderate/severe in terms of replacement route. For patients with multiple recorded episodes of hypomagnesemia during their hospital stay, only the initial occurrence was included in the analysis. Those with hypermagnesemia were not included.

Detection of acute cognitive decline

Electronic hospital records were reviewed thoroughly and individually for each patient to identify episodes of acute cognitive decline, defined in this study as any acute worsening from baseline cognitive function. These included cases of acute delirium which encompassed the hyperactive, hypoactive, and mixed subtypes, sudden deterioration of pre-existing cognitive impairment regardless of its underlying etiology, and new or increased confusion. The majority of patients were assessed using the 4-AT screening tool [9], with scores of four or higher indicating probable delirium, coupled with collateral history. The 4AT Rapid Clinical Test for Delirium Detection encompasses four components, namely Alertness, Abbreviated Mental Test-4 (assesses age, date of birth, name of hospital/building, current year), Attention and Acute change or fluctuating course. Those without a 4-AT score documented were validated through detailed clinical documentation and corroborating collateral history. For those with established cognitive disorders such as dementia, acute cognitive decline was determined through careful review of the extensive collateral history provided by partners, relatives, friends, and formal caregivers to distinguish acute changes from chronic cognitive patterns. To ensure consistency in identification, three reviewers assessed the multidisciplinary notes of each case, including admission clerking, nursing notes, and daily medical reviews, and identified documented clinical language such as ‘delirium’, ‘new confusion’, ‘acute confusion’, ‘new-onset cognitive change’, ‘new cognitive impairment’, ‘increased confusion on background of underlying cognitive impairment’ alongside 4-AT results where available. Cases where documentation was unclear were conservatively classified as not having cognitive decline to minimize the risk of overestimation. Where discrepancies among reviews occurred, cases were discussed and consensus was reached among the reviewers.

Statistical analyses

All statistical analyses were performed using R version 4.5.1 (R Core Team, R Foundation for Statistical Computing, Vienna). Continuous variables were summarized as mean ± standard deviation, while categorical variables were expressed as frequencies and percentages. Data analyses were conducted using independent t-tests for normally distributed continuous variables, and chi-square tests for categorical variables. All relevant statistical assumptions were checked and deemed appropriate for the methods applied.

To assess the association between magnesium levels and acute cognitive decline while controlling for potential confounders, multivariable logistic regression was performed, which adjusted for age, sex and nine prevalent acute confounders in the final analyses. These potential confounders included infection, electrolyte disturbances (i.e., hypocalcemia, hypercalcemia, hyponatremia and hypernatremia), acute kidney injury, pain, acute stroke, and constipation. These confounders were selected as they are commonly encountered clinical factors known to contribute to acute cognitive impairment in old and frail patients. Adjusting for these common clinical drivers is essential to ensure the observed association between hypomagnesemia and acute cognitive decline is as credible and independent as possible. Several other acute confounders known to adversely affect cognitive outcomes, as mentioned in the results section, were also initially considered to comprehensively account for potential contributors to acute cognitive decline, but were excluded in the final model due to infrequent occurrence within this specific cohort (<2% incidence) or absence in certain groups. This is to mitigate the risk of statistical instability.

Odds ratios (ORs) with 95% confidence intervals (CIs) were calculated to quantify effect sizes. Model fit was evaluated using the Akaike Information Criterion (AIC) and Bayesian Information Criterion (BIC). A p-value of <0.05 was considered statistically significant for all analyses. This approach ensured appropriate adjustment for demographic and clinical variables while examining the independent relationship between magnesium status and acute cognitive decline. The logistic regression model accounted for multiple covariates, including infection, electrolyte disturbances (hypocalcemia, hypercalcemia, hyponatremia, hypernatremia), acute kidney injury, pain, acute stroke, and constipation.

## Results

Adherence to treatment and monitoring guidelines

Among the 667 hospitalised old and frail patients included in this audit, 149 (22.3%) were found to have hypomagnesemia (serum magnesium ≤0.69 mmol/L) on at least one occasion during their admission, whilst the remaining 518 patients (77.7%) had normal magnesium levels during their hospital stay.

For treatment, the trust recommends oral magnesium replacement (typically magnesium aspartate sachets) for mild cases and intravenous supplementation with magnesium sulfate for moderate-to-severe deficiency or patients unable to tolerate oral therapy. For monitoring, the guideline recommends daily monitoring until levels improve, then three times weekly until normal for mild and moderate cases, and 12-hourly monitoring for severe hypomagnesemia.

The audit identified significant room for improvement in adherence to both treatment and monitoring guidelines for hypomagnesemia. Only 56 of the 149 patients with documented hypomagnesaemia (37.6%) received any form of magnesium replacement therapy. Among 149 patients with hypomagnesemia, 18 (12.1%) presented with moderate-to-severe deficiency (≤0.5 mmol/L). Of these, intravenous magnesium sulfate was administered to five patients (27.8%), while seven (38.9%) received oral replacement with either magnesium aspartate sachets or magnesium glycerophosphate chewable tablets, and six (33.3%) received no magnesium supplementation. Among 131 patients with mild hypomagnesemia, 45 (34.4%) received either oral or intravenous supplementation, while 86 (65.6%) received no treatment. As for monitoring, follow-up magnesium testing was performed within the guideline timeframes for only 60 cases (40.3%), irrespective of their treatment status. The results are summarized in Table 1.

**Table 1:** Summary of treatment and monitoring practices for 149 patients with documented hypomagnesemia including the route of replacement

Parameter	n/N (%)
All hypomagnesemia cases	149/667 (22.3%)
Received any replacement	56/149 (37.6%)
Moderate-severe (≤0.5 mmol/L)	18/149 (12.1%)
IV replacement	5/18 (27.8%)
Oral replacement	7/18 (38.9%)
No treatment	6/18 (33.3%)
Mild (0.5-0.69 mmol/L)	131/149 (87.9%)
IV or oral replacement	45/131 (34.4%)
No treatment	86/131 (65.6%)
Monitoring adherence	60/149 (40.3%)

Association between hypomagnesemia and acute cognitive decline

The study cohort comprised 667 hospitalized elderly patients (283 male patients, 384 female patients) with a mean age of 82.14±9.29 years (range 55-100). Patients were categorized into four sub-groups by their magnesium status and the presence of acute cognitive decline: 362 (54.3%) had normomagnesemia (0.7-1.0 mmol/L) without acute cognitive decline; 72 (10.8%) exhibited hypomagnesemia (≤0.69 mmol/L) but preserved baseline cognition; 156 (23.4%) had normomagnesemia alongside acute cognitive decline; and 77 (11.5%) presented with concurrent hypomagnesemia and acute cognitive decline. Results are summarized in Table 2.

**Table 2:** Demographic characteristics of patients stratified by their magnesium status and presence of acute cognitive decline Patients were categorized into four subgroups based on magnesium levels and cognitive status. Group characteristics are presented by age and sex distribution.

Group	n (% total)	Age, mean (SD)	Male patients, n (%)	Female patients, n (%)
Normomagnesemia, no acute cognitive decline	362 (54.3%)	81.7 (9.2)	155 (42.8%)	207 (57.2%)
Hypomagnesemia, no acute cognitive decline	72 (10.8%)	78.7 (12.0)	30 (41.7%)	42 (58.3%)
Normomagnesemia, acute cognitive decline	156 (23.4%)	84.5 (8.1)	72 (46.2%)	84 (53.8%)
Hypomagnesemia, acute cognitive decline	77 (11.5%)	82.6 (8.1)	26 (33.8%)	51 (66.2%)
Total	667	82.1 (9.3)	283	384

The analysis adjusted for age, sex, and clinically significant acute confounders, including infection (52.3%, 349/667), electrolyte disturbances (hypocalcemia, hypercalcemia, hyponatremia, hypernatremia), acute kidney injury, pain, acute stroke, and constipation. Additional acute confounders such as new or progressive space-occupying lesions, post-seizure states, hepatic encephalopathy, thyroid disorder, alcohol use, new normal pressure hydrocephalus, and high-risk medications such as steroids, benzodiazepines, anticholinergics, and opioids were initially considered but excluded in the final model due to infrequent occurrence within the cohort (<2% incidence) or absence in key comparison groups, which caused regression instability and did not result in meaningful adjustment. The results are summarized in Table 3.

**Table 3:** Distribution of acute clinical confounders by magnesium (Mg) status and cognitive outcome Comparison of key acute confounding variables across patients with and without hypomagnesemia and cognitive decline.

Confounder	No acute cognitive decline		Acute cognitive decline		Total
	Low Mg (n=72)	Normal Mg (n=362)	Low Mg (n=77)	Normal Mg (n=156)	(N=667)
Acute stroke	10 (13.9%)	27 (7.5%)	1 (1.3%)	1 (0.6%)	39 (5.8%)
Infection	42 (58.3%)	186 (51.4%)	36 (46.8%)	85 (54.5%)	349 (52.3%)
Hypocalcemia	3 (4.2%)	0 (0.0%)	8 (10.4%)	3 (1.9%)	14 (2.1%)
Hypercalcemia	1 (1.4%)	2 (0.6%)	2 (2.6%)	11 (7.1%)	16 (2.4%)
Hyponatremia	2 (2.8%)	8 (2.2%)	10 (13.0%)	10 (6.4%)	30 (4.5%)
Hypernatremia	0 (0.0%)	3 (0.8%)	5 (6.5%)	7 (4.5%)	15 (2.2%)
Acute kidney injury	15 (20.8%)	33 (9.1%)	16 (20.8%)	27 (17.3%)	91 (13.6%)
Dehydration	0 (0.0%)	4 (1.1%)	13 (16.9%)	8 (5.1%)	25 (3.7%)
Pain	3 (4.2%)	77 (21.3%)	0 (0.0%)	15 (9.6%)	95 (14.2%)

In the multivariable logistic regression model adjusting for age, sex, and relevant clinical confounders, hypomagnesemia was independently associated with significantly increased odds of acute cognitive decline. After adjusting for the selected confounders, patients with hypomagnesemia had 2.35 times greater odds of developing acute cognitive deterioration compared to those with normal magnesium levels. Increasing age was also associated with a higher risk. Electrolyte disturbances, including hypercalcemia, hyponatremia, and hypernatremia, were also significantly associated with increased odds of acute cognitive decline. Constipation demonstrated a similar association. In contrast, acute stroke and pain were associated with reduced odds of cognitive decline, which may reflect diagnostic overshadowing or differences in documentation rather than true protective effects. Other variables, including sex, infection, hypocalcemia, and acute kidney injury, did not demonstrate statistically significant associations in the adjusted model. The results are summarized in Table 4.

**Table 4:** Multivariable logistic regression of factors associated with acute cognitive decline in the hospitalized old and frail patients, showing adjusted odds ratios (95% CIs) Reference groups: normal magnesium, female sex, and absence of each comorbidity; Model fit: Akaike information criterion (AIC)=772.9 and Schwarz's Bayesian Information Criterion (BIC)=831.5

Variable	Category	OR (95% CI)	p-value	Standard error (SE)	Test statistic (Z-value)
Magnesium status	Normal	Reference	-	-	-
	Low	2.35 (1.54–3.60)	<0.001	0.216	3.962
Sex	Female	Reference	-	-	-
	Male	1.32 (0.92–1.91)	0.133	0.186	1.504
Age (per year)		1.04 (1.02–1.06)	<0.001	0.010	3.691
Infection		0.90 (0.63-1.30)	0.583	0.183	-0.549
Hypocalcemia		2.81 (0.81-13.04)	0.131	0.684	1.512
Hypercalcemia		8.64 (2.43–41.24)	0.002	0.700	3.082
Hyponatremia		3.45 (1.54–8.15)	0.003	0.421	2.941
Hypernatremia		5.64 (1.58–26.55)	0.013	0.697	2.482
Acute kidney injury		1.27 (0.76-2.09)	0.350	0.256	0.934
Constipation		2.53 (1.18–5.48)	0.017	0.389	2.388
Pain		0.38 (0.20–0.69)	0.002	0.314	-3.058
Acute stroke		0.12 (0.02–0.40)	0.004	0.745	-2.811

## Discussion

This study demonstrated that hypomagnesemia may be a significant and independent contributor to acute cognitive decline in this cohort of old and frail patients. After adjusting for age, sex, and a range of clinically relevant acute confounders, low serum magnesium was associated with more than twice the odds of developing cognitive deterioration compared to normal magnesium levels. These findings appear to be aligned with the growing body of evidence suggesting that magnesium plays a critical and under-recognized role in neurological resilience, particularly in older adults.

Magnesium plays a crucial neuroprotective role by acting as a natural N-methyl-D-aspartate (NMDA)‑receptor blocker, which helps to prevent calcium-induced neuronal damage. When magnesium levels drop, NMDA receptors can become overactive, leading to neuronal excitotoxicity, which is a key mechanism implicated in delirium and sudden cognitive decline [10]. Additionally, magnesium deficiency fuels inflammation and oxidative stress by increasing the production of pro-inflammatory cytokines like IL‑1 and TNF‑α, and ramps up reactive oxygen species while impairing antioxidant defenses. These effects are particularly detrimental to older adults, who have increased blood-brain barrier permeability and reduced mitochondrial resilience. Finally, magnesium is essential for synaptic plasticity and efficient energy metabolism within the brain. Hence, deficiencies can compromise both memory function and cognitive reserve [10]. This is also consistent with the study by Hansen et al., which highlighted that hypomagnesemia is a common and clinically significant electrolyte disturbance in critically-ill patients, contributing to the development of delirium through mechanisms such as increased neuronal excitability and impaired synaptic function [11]. In another study, hypomagnesemia was associated with increased neuromuscular excitability, presenting as delirium and movement disorders such as tremors, tetany and dystaxia [12]. Patel et al. also emphasized that magnesium plays a vital role in regulating neurobiological processes by suppressing Nuclear factor kappa-light-chain-enhancer of activated B cells (NF-κB) activation, which underlies its strong anti-inflammatory properties, thereby maintaining neuronal integrity [13].

In a geriatric ward study, hypomagnesemia was associated with a 5.8-fold increase in delirium risk (95% CI: 1.45-23.2; p=0.013), even after adjusting for age, Mini-Mental State Examination (MMSE), renal function, and diuretic use [3]. A retrospective ICU study found that hypomagnesemia at admission doubled the risk of delirium (adjusted hazard ratio (HR) 2.12; 95% CI 1.03-4.38), independent of sedative use, BMI, and immunosuppressants [5]. In a pilot trial, adding intravenous magnesium to sedation protocols increased delirium-free days (from 55% to 89%), indicating a protective effect on acute brain dysfunction [14]. Importantly, the association between hypomagnesemia and acute cognitive decline remained significant even after adjusting for other electrolyte disturbances, acute kidney injury, and acute clinical presentations such as infection, pain, and stroke.

Some findings, such as acute stroke and pain appearing protective against acute cognitive decline, may seem counterintuitive. However, this likely reflects the trust’s clinical structure and admission patterns. Patients labelled with acute stroke were mainly managed in the stroke rehabilitation ward within the geriatrics department. The rehabilitation is focused on neurological recovery, with differing care frameworks and documentation. Acute cognitive decline is also hard to detect in this group of patients due to expressive/receptive dysphasia and dysarthria. We are unsure why pain appeared protective against acute cognitive decline in our findings. However, possible explanations include the clinical characteristics and selection criteria of patients admitted under the orthogeriatric subunit. These patients are typically stable, relatively fit, and selected for well-defined surgical or conservative management following trauma-related bony injuries. They often have few active comorbidities, and the focus of care is primarily on rehabilitation. Furthermore, patients with documented pain are by necessity able to verbalize and express their symptoms, which is a capacity less likely in those acutely confused. Thus, these protective associations likely reflect differences in admission intent and care framework, not a true inverse relationship.

The study also revealed suboptimal adherence to local treatment and monitoring protocols for hypomagnesemia. Despite clear institutional guidelines, fewer than 40% of patients with documented hypomagnesemia received any replacement therapy, and only 40.3% underwent appropriate follow-up testing. These gaps in care may allow correctable cognitive decline to go unrecognized and untreated, especially in frail or clinically complex patients where acute cognitive decline can easily be misattributed to other causes or dismissed as baseline confusion.

There are several possible reasons why adherence to hypomagnesemia management guidelines may be suboptimal in clinical practice. First, magnesium is often perceived as a less critical electrolyte compared to sodium, potassium, or calcium, which may lead to its under-prioritization, particularly in busy ward environments. Second, symptoms of magnesium deficiency can be non-specific or masked by other clinical issues, making it less likely to be recognized as a treatment priority. Additionally, some clinicians may not be fully aware of the cognitive consequences associated with low magnesium, especially in frail older patients. Practical barriers may also contribute, such as uncertainty around appropriate replacement dosing, lack of familiarity with the guideline itself, or limited prompts within electronic prescribing systems to support timely rechecking after treatment. Together, these factors may result in missed opportunities to identify and correct magnesium deficiency, which can potentially improve cognitive outcomes.

This study was designed to investigate the impact of hypomagnesemia on the acute cognitive function of the old and frail patients in the trust. Accordingly, all confounders included in the analysis reflected acute physiological stressors relevant to the admission, such as infection, metabolic disturbances, medication burden, and renal dysfunction. Pre-existing or longer-term cognitive impairment was intentionally excluded as a confounder in this study as it represents a chronic condition with a complex, potentially confounded relationship to both exposure and outcome. It may share upstream causes with hypomagnesemia, such as frailty and poor nutrition, or lie on the causal pathway if chronic magnesium depletion contributes to neurodegeneration [15]. Previous studies have identified strong correlation between low magnesium levels and dementia [16]. Adjusting for dementia could therefore introduce confounding bias and obscure the independent role of magnesium deficiency in acute cognitive change. Including it would also require parallel adjustment for a wide range of other chronic vulnerabilities, which was beyond the scope and design of this analysis.

These findings carry important clinical implications. Cognitive deterioration in older adults is associated with prolonged hospital stays, higher risk of complications, and long-term functional decline [3,5]. As magnesium deficiency may be an easily modifiable contributor to these outcomes, it deserves greater clinical attention. Simple interventions such as timely magnesium replacement and appropriate monitoring may represent a cost-effective strategy to preserve neurological function and quality of life in the vulnerable population of the hospital.

Limitations 

This study was designed to evaluate clinical practice and outcomes within a single NHS Trust, where patient selection, documentation standards, and care delivery are shaped by local policies, organization structures, frameworks of care, and electronic record systems. As such, the findings may not be directly generalizable to other settings.

The retrospective nature of the study also presents inherent limitations, including the potential for incomplete or inconsistent documentation, particularly regarding cognitive assessments and the recognition of delirium. Although the 4-AT screening tool was used in most cases, it was not universally documented in the medical records. In cases without a recorded 4-AT score, acute cognitive decline was identified through detailed review of clinical notes and collateral history. While this approach reflects routine clinical practice, it may have introduced some variability in case identification despite efforts to apply consistent criteria during data extraction. Furthermore, the retrospective design also poses a challenge in inferring temporal directionality, i.e., whether hypomagnesemia preceded or followed acute cognitive decline.

Missing data, particularly with regard to follow-up magnesium levels and cognitive assessments, were handled through case-wise exclusion. No imputation methods were applied. Although the precise proportion of missing data was not formally quantified, it was estimated to be low and unlikely to affect the validity of the findings.

Recommendations

This evaluation can inform future studies in exploring the trajectory of cognitive recovery following magnesium repletion and stratified analyses that compare outcomes between patients with and without underlying neurocognitive disorders. Larger, prospective studies may also help determine whether routine magnesium monitoring should be standard in older patients presenting with confusion, particularly in the absence of other clear causes.

Moreover, we recommend incorporating clinical decision support prompts within the electronic health record to facilitate timely monitoring and rechecking of serum magnesium levels in high-risk patients, particularly those presenting with acute cognitive changes.

## Conclusions

This evaluation identified notable variation in the management of hypomagnesemia among older inpatients within this NHS Trust, with limited adherence to existing treatment and monitoring guidelines. In the cohort reviewed, patients with low serum magnesium appeared more likely to experience acute cognitive decline that led to or developed during admission, even after adjusting for other acute factors such as infection and electrolyte disturbances.

While the purpose of this evaluation was not to establish causation, the findings suggest that greater consistency in identifying and managing hypomagnesemia may represent an opportunity to improve care for old and frail patients. Strengthening adherence to local protocols could be highlighted as a cost-effective strategy to help reduce the risk of avoidable complications such as delirium and support better cognitive outcomes in this vulnerable population.
